# Production and purification of ^43^Sc and ^47^Sc from enriched [^46^Ti]TiO_2_ and [^50^Ti]TiO_2_ targets

**DOI:** 10.1038/s41598-023-49377-7

**Published:** 2023-12-19

**Authors:** Shelbie J. Cingoranelli, Jennifer L. Bartels, Pavithra H. A. Kankanamalage, C. Shaun Loveless, David A. Rotsch, Suzanne E. Lapi

**Affiliations:** 1 Department of Chemistry, University of Alabama at Birmingham, 1924 6th Ave. S., WTI 310F, Birmingham, AL 35244 USA; 2 Department of Radiology, University of Alabama at Birmingham, Birmingham, USA; 3 Physics Division, Argonne National Laboratory, Lemont, USA; 4 Radioisotope Science and Technology Division, Oak Ridge National Laboratory, Oak Ridge, USA

**Keywords:** Nuclear chemistry, Positron-emission tomography

## Abstract

The radioscandium isotopes, ^43^Sc and ^47^Sc, compose a promising elementally matched theranostic pair that can be used for the development of imaging and therapeutic radiopharmaceuticals with identical structures. This study aimed to investigate the production of high radionuclidic purity ^43^Sc from enriched [^46^Ti]TiO_2_ targets and ^47^Sc from enriched [^50^Ti]TiO_2_ targets and establish a target recycling technique. Enriched [^46^Ti]TiO_2_ targets were irradiated with 18 MeV protons, and enriched [^50^Ti]TiO_2_ targets were bombarded with 24 MeV protons. ^43^Sc and ^47^Sc were purified using ion chromatography attaining recovery yields of 91.7 ± 7.4% and 89.9 ± 3.9%, respectively. The average radionuclidic purity for ^43^Sc was 98.8 ± 0.3% and for ^47^Sc 91.5 ± 0.6%, while the average recovery of enriched titanium target material was 96 ± 4.0%. The highest apparent molar activity for [^43^Sc]Sc-DOTA was 23.2 GBq/µmol and 3.39 GBq/µmol for [^47^Sc]Sc-DOTA. This work demonstrates the feasibility of using enriched recycled [^46^Ti]TiO_2_ and [^50^Ti]TiO_2_ targets to produce high purity ^43^Sc and ^47^Sc as an elementally matched theranostic isotope pair.

## Introduction

Theranostic techniques empower physicians to make decisions for targeted therapy based on imaging obtained using a similar or identical construct^1–4^. This approach involves the development of two chemically similar or identical radiopharmaceuticals, each serving a distinct purpose: one as a diagnostic agent and the other as a therapeutic agent. The key distinction lies in the incorporation of different radioisotopes. Diagnostic radiopharmaceuticals utilize radioisotopes with suitable decay properties, emitting detectable photons of energy that can escape the patient whereas therapeutic radiopharmaceuticals employ radioisotopes that emit β-, Auger electrons, or α particles, capable of causing DNA strand breaks.

Theranostic strategies leverage these distinct decay properties to create chemically similar drugs with different purposes^1^. The theranostic pairing in several FDA-approved radiopharmaceuticals employs ^68^Ga and ^177^Lu for targeted imaging and therapy in prostate and neuroendocrine cancers and has generated an increased interest in this technique. A disadvantage of using this pair is that ^68^Ga and ^177^Lu are chemically different, which may result in different pharmacokinetics of the resulting radiopharmaceuticals^2–7^. The ideal theranostic pair would include radioisotopes of the same element but with different emissions (i.e., one suitable for diagnosis and the other for therapy).

The radioisotopes of scandium can provide a true elementally matched pair for theranostic use^8,9^. Two positron (β^+^) emitting radioscandium isotopes, ^43^Sc (β^+^ = 476 keV; t_1/2_ = 3.89 h) and ^44g^Sc (β^+^ = 632 keV, t_1/2_ = 3.97 h), are suitable for Positron Emission Tomography (PET) imaging of small molecules and peptides. These positron-emitting radioscandium nuclides serve as chemically identical radiopharmaceuticals to the ones radiolabeled with therapeutic ^47^Sc. The therapeutic ^47^Sc (β^−^ = 160 keV; t_1/2_ = 3.34 days) can also be employed in Single Photon Emission Computed Tomography (SPECT) due to the emission of a 159 keV γ-ray, which can be used to estimate dosimetry and visualize the biodistribution using SPECT imaging^10^. One of the main advantages of radioscandium nuclides is their ability to create a true elementally matched theranostic pair. This stems from their shared chemistry, including separation, purification, and radiolabeling conditions, resulting in identical in vivo kinetics and dynamics of these compounds. Utilizing the same element for either diagnostic (^43^Sc and ^44^Sc) or therapeutic (^47^Sc) compounds presents a significant advantage over the currently used ^68^Ga/^177^Lu pair. Table S1 summarizes the relevant characteristics of ^68^Ga, ^177^Lu, and ^43,44g,47^Sc.

Recent literature has shown an increased interest in the production and purification of radioscandium isotopes^5,11^. Several production routes are being investigated, including proton, deuteron, alpha, neutron, and photon bombardment using calcium (Ca) and titanium (Ti) target material^12–16^. However, a significant challenge with these production routes is the co-production of other radioscandium isotopes from the multiple stable isotopes of Ca and Ti, with the long-lived ^46^Sc being the primary contaminant of concern^17^.

Five naturally occurring stable isotopes of Ti can be exploited to produce radioscandium from proton irradiation. The four main proton-induced reactions to produce medically interesting radioscandium isotopes are ^46^Ti(p,α)^43^Sc, ^47^Ti(p,α)^44^Sc, ^48^Ti(p,2p)^47^Sc, and ^50^Ti(p,α)^47^Sc. A challenge behind these production routes is the natural abundances of Ti isotopes (^46^Ti: 8.25%; ^47^Ti: 7.44%; ^48^Ti: 73.72%; ^49^Ti: 5.41%; ^50^Ti: 5.18%) that lead to the co-production of multiple radioscandium nuclides when non-enriched material is used. Table S2 contains all the radioscandium nuclides produced via irradiation of natural abundance titanium targets, their respected nuclear reactions, half-lives, decay mode and characteristic γ-gamma rays used for their identification and activity quantification. Enriched material will result in lower production of the undesired radioscandium nuclides^17–19^. The impurities listed in Table S2 serve as a reminder of the challenges in achieving high purity radioisotopes, which can be partially addressed using enriched material. Target material that is highly enriched in one titanium isotope is a viable option, as the lower abundance of the titanium isotopes will decrease the production of the radioisotopic impurities.

This study aimed to investigate the production of ^43^Sc from enriched [^46^Ti]TiO_2_ and ^47^Sc from enriched [^50^Ti]TiO_2_ targets and to develop a reliable recycling method for the target material to offset the enriched target material cost. Quantification of yields and radionuclide purification utilized High Purity Germanium (HPGe) analysis and trace metal analysis using Inductively Coupled Plasma-Mass Spectrometry (ICP-MS). The quality of the products was further examined using a 1,4,7,10-tetraazacyclododecane-1,4,7,10-tetraacetic acid (DOTA) titration. PET imaging of a ^43^Sc phantom was also carried out.

## Materials and methods

### Reagents

All chemicals used were analytical or trace metal grade unless otherwise stated. Water was obtained from a deionized 18.2 MΩ-cm Milli-Q System (Millipore, Billerica, MA). Hydrochloric acid (HCl, 37% by weight, 99.999%), ammonium bifluoride (NH_4_HF_2_, 99.999%), and 28% ammonia solution (NH_4_OH, 99.999%) were purchased from Sigma-Aldrich (St. Louis, MO). Nitric acid (HNO_3_, 67–70% by weight, 99.999%) was purchased from Fisher Scientific (Hampton, NH, USA). Enriched ^46^Ti and ^50^Ti were provided by the National Isotope Development Center, USA. The isotopic percentage, provided by the National Isotope Development Center, is given in Table S3 in supplemental information and the Certificates of Analysis in Table S4. The titanium cover foil with 4N5 purity and tantalum coin backing with 3N8 purity were purchased from ESPI Metals (Ashland, OR, USA). All periodic table analytical standards (mix 101, 103, 104, 5% HNO_3_, 10 mg/L) and SG-iTLC plates were purchased from Agilent Technologies (Santa Clara, CA, USA). Analytical-grade *N,N,N′,N′-*tetra-2-ethylhexyldiglycolamide (branched DGA) resin was purchased from Eichrom Technologies (Lisle, IL) and empty 1 mL SPE fritted columns, 0.2 µm additional frits, and column adaptors were purchased from Sigma-Aldrich (Supelco, Sigma-Aldrich, St. Louis, MO). DOTA was purchased from Macrocyclics (Plano, TX, USA). The Micro Deluxe Phantom™ with the Micro Deluxe Cold Rod Insert™ was purchased from Data Spectrum Corporation (Durham, NC, USA). Conical 15 mL screwcap perfluoroalkoxy alkane (PFA) digestion vessel, 6 mL Octagonal Body Vial, and PFA 10 mL volumetric flasks were purchased from Savillex (Eden Prairie, MN). Millipore Sigma MF-Millipore Mixed Cellulose Ester Membranes with 0.22 µm pore size were purchased from Fisher Scientific (Hampton, NH, USA). The ^68^Ga was eluted from Eckert and Ziegler ^68^Ge/^68^Ga generator using 0.1 M HCl and was concentrated on an Agilent Bond Elute SCX cartridge, where ^68^Ga was recollected in 250 µL 5 M NaCl/0.1 M HCl. All glassware was cleaned in a 20% HNO_3_ bath overnight before use.

### Target preparation and irradiation conditions

The 2-mm thick tantalum (Ta) target coins and target material preparation were carried out as previously reported by Loveless et al.^17^. Briefly, the TiO_2_ material was kept at 250 °C in an oven for at least 24 h before bombardment. Approximately 100 mg targets were pressed in a FTIR pellet 10-mm evacuable die (Specac, Kent, UK) by increasing the applied pressure in 1-ton increments per minute up to 5 tons, then held at 5 tons for 15 min using a Carver model 3664 hydraulic press (Carver, INC, Wabash, IN, USA). The pellet was removed and placed into the divot of a 2 mm Ta coin. Targets were irradiated on a TR-24 cyclotron (Advanced Cyclotron Systems, Inc., Richmond, BC, Canada) using a 90° coin target holder (Advanced Cyclotron Systems, Inc., Richmond, BC, Canada)^20^. For ^43^Sc production using [^46^Ti]TiO_2_, targets were bombarded with 18 MeV protons at 20 µA for 1.5 h. For ^47^Sc production using [^50^Ti]TiO_2_, targets were bombarded with 24 MeV protons at 20 µA for 4 or 8 h. The theoretical predicted activity was calculated based on the cross section measurements retrieved from EXFOR shown in Fig. S1^21–23^. Cross sections for additional co-produced radiocontaminants are presented in the supplemental information Fig. S2.

### Target digestion and purification

#### Digestion of TiO_2_

The irradiated target material was removed from the Ta coin and added to a 15-mL screwcap PFA digestion vial with 300 mg of NH_4_HF_2_. The vial was capped and placed into a furnace at 250 °C for 2 h. After digestion, the vial was removed and allowed to cool before adding 5 mL of concentrated HCl. The vial was heated in a silicone oil bath at 160 °C for 45 min. The dissolved solution was transferred into a 10 mL PFA volumetric flask and diluted to 10 mL, using 1 mL additions of 9 M HCl, rinsing the digestion vial in the process for a final concentration of 10.5 M HCl.

#### Purification

Approximately 150 mg of branched DGA resin was added to an empty SPE column with frits on either side of the resin bed. The column was conditioned with a syringe pump at 2 mL/min with the following solutions: 20 mL of 7 M HNO_3_, 20 mL of 1 M HNO_3_, 20 mL of Milli-Q water, 20 mL of 0.1 M HCl and 20 mL of 9 M HCl. Air was pushed through after each solution. Following column conditioning, the dissolved target was loaded onto the column through a 10 mL syringe, which was either pushed manually or by using a syringe pump with the flow rate set to 2 mL/min (collected in Flow-Through (FT) tube). The column was then washed with 20 mL of 9 M HCl (Elution 1 (E1)), followed by 10 mL of 7 M HNO_3_ (Elution 2 (E2)), 3 mL of 1 M HNO_3_ (Elution 3 (E3)), and three separate, 3 mL additions of 0.1 M HCl (Elution 4-6 (E4-6)) as shown in Fig. S3. Each of the eluents was collected in individual Falcon tubes. The E4 fraction was evaporated to dryness using a Smart Evaporator (BioChromato) before being reconstituted with 200 μL 0.1 M HCl and used for further studies. The evaporation conditions used a 5 mL PFA vial, heated at 100 °C in an Al bead bath under vacuum without the use of N_2_.

#### Target material recycling

The FT and E1 fractions were combined and diluted to 500 mL with Milli-Q water in a 1 L glass beaker. The beaker was wrapped with plastic wrap and the solution was heated to 80 °C for 1 h and then allowed to cool. While stirring, the solution was adjusted to pH 8 with 1 mL additions of 28% ammonium hydroxide, until the TiO_2_ precipitated as a white cloudy solution. The solution/precipitate was left to settle overnight at room temperature and then vacuum filtered using a 0.22 μm mixed cellulose filter paper on a low vacuum. After the filter paper was dried, the TiO_2_ was collected into a clean, dry beaker and placed into a vacuum furnace at 250 °C for at least 24 h before the next bombardment.

#### Gamma-ray spectroscopy

Gamma-ray spectroscopy was used for isotope identification, to determine radionuclide yields and radionuclidic purity using a Canberra GC2018 High Purity Germanium detector (HPGe), interfaced with a DSA = 100 multichannel analyzer (Meriden, CT, USA). Data acquisition and analysis were performed using Genie 2000 software (Canberra). A 1.5 mL microcentrifuge mixed, sealed, source, 8303-EG-SD (Eckert and Ziegler Analytics, Atlanta, GA, USA) was used for energy and efficiency calibration. The radionuclides within the source were ^57^Co(1.23 MBq)), ^60^Co(2.50 MBq) ^88^Y(4.86 MBq), ^85^Sr(3.61 MBq), ^109^Cd (5.41 MBq), ^113^Sn(3.09 MBq), ^137^Cs(1.31 MBq), and ^139^Ce(1.83 MBq). One and 10 μL aliquots of samples were diluted to 1 mL with MilliQ water and were analyzed at distances of 5 mm or 25 mm from the bottom of the tube to the face of the HPGe detector. Equations (1) and (2) below were used to determine end-of-bombardment yields:

1 $$A_{1} = \frac{{\left( {N_{p} *\lambda } \right)}}{{\left( \varepsilon \right)\left( {1 - e^{{ - \lambda t_{1} }} } \right)\left( {1 - D_{r} } \right)\left( {I_{\gamma } } \right)}}$$

2 $$A_{o} = \frac{{A_{1} }}{{e^{{ - \lambda t_{o} }} }}$$

where A_1_ is the activity calculated from the HPGe acquisition, N_p_ is the net peak area in each photopeak, λ is the decay constant of radioisotope of interest, ε is the detector efficiency of the photopeak, t_1_ is the real-time of HPGe acquisition, D_r_ is the average dead time of the instrument, I_γ_ is the branching ratio of the γ-ray of the radioisotope, A_o_ is the activity at the end of bombardment, and t_o_ is the time passed between the end of bombardment and the time of acquisition^24^. Measurements were taken for a minimum of 500 counts under each photopeak and a deadtime no larger than 5%.

#### Inductively coupled plasma mass spectrometry (ICP-MS)

Elemental analysis was performed on Agilent Technologies 7800 ICP-MS (Santa Clara, CA, USA) with Agilent software, ICP-MS MassHunter v4.3. A 20 µL aliquot from each fraction from the separation was diluted to 10 mL with 2% HNO_3_ to determine the elements present, in triplicate. The dissolved target solution and the flow-through collection had a secondary dilution step where 400 µL of the first ICP-MS sample was diluted to 10 mL in 2% HNO_3_. Multi-element standards in 2% HNO_3_ were used for calibration of 0.1, 0.5, 1, 5, 10, 100, 200, 400, 600, 800, and 1000 ppb for the calibration curve and prepared in 10 mL volume from a 10 µg/mL stock. Elements selected for monitoring are given in the supplemental information Table S5.

#### Apparent molar activity

To determine apparent molar activity (AMA), a DOTA titration was performed using ^43^Sc or ^47^Sc, after which, the half-maximum effective concentration (EC_50_) for complete complexation was determined by taking the best-fit values of a transform of the log(µmol) versus percent radiolabeled, performed using Prism 8 software. Then, the average activity added was divided by EC_50_ multiplied by 2, shown in Eq. (3) below.

3 $$AMA = \frac{{\text{Average activity}}}{{\left( {EC_{50} \times 2} \right)}}$$

Details of the titration are provided in the supplemental information; section i of the supplemental and Table S6. Analysis of the samples was performed via instant thin-layer chromatography by spotting 1 µL of sample on an iTLC-SG paper and developing in 1 M citrate buffer. An Eckert & Ziegler AR2000 TLC scanner (Berlin, Germany) was used for TLC analysis.

#### Phantom imaging

A phantom composed of poly(methyl methacrylate) with rod diameters between 1.2 and 4.8 mm was used for imaging studies. Complete details of the phantom dimensions are provided in supplemental information Fig. S5. The preparation process for the phantoms containing ^18^F, ^43^Sc, or ^68^Ga involved diluting each radioactive solution to 20 mL with Milli-Q water (MQ). Subsequently, 18 mL of the diluted solution was added to the phantoms to achieve a radioactivity of 3.72 ± 0.06 MBq (100.6 ± 1.7 µCi). The phantoms were scanned for 30 min on the UAB small animal GNEXT PET/CT scanner (GNEXT PET/CT, Sofie Biosciences, CA, USA), with an energy window of 350–650 keV, followed by a 5-min CT scan at a voltage 80 kVp, current 150 μA, and 720 projections. Images were reconstructed using a 3D-OSEM (Ordered Subset Expectation Maximization) algorithm (24 subsets and 3 iterations, with random, attenuation, and decay correction). Additionally, the ^18^F and ^43^Sc were reconstructed with the same number of total decays as the ^68^Ga scan (1693 s for ^18^F and 1614 s for ^43^Sc). The images were processed using the description by Bunka et al.^25^. Image analysis was performed using VivoQuant software (VivoQuant 4.0, Invicro Imaging Service and Software, Boston USA), where one representative transversal section was used and analyzed at the different depths of the phantom. The resulting intensity plots of each phantom were exported to Origin^*^ 2022 (OriginLab), where the full-width at half-maximum (FWHM) was processed for each slice, and an arithmetic mean and standard deviation were obtained^25,26^. All three radionuclides’ FWHM were compared for all visibly distinguishable rods. The ^43^Sc FWHM were statistically compared to ^18^F and ^68^Ga FWHM using either one-way ANOVA, on rods 4.8, 4.0 and 3.2 mm, or to ^18^F only using t-test statistics for 2.4 and 1.6 mm rods.

## Results

### Digestion and separation yields

**Figure 1:**
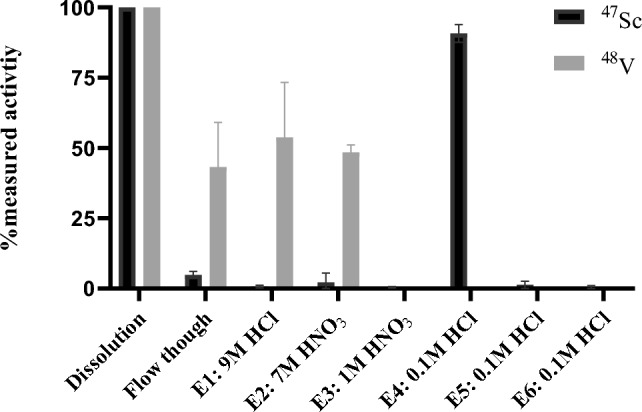
The elution profile of the ^47^Sc separation from enriched [^50^Ti]TiO_2_ targets, shown as a percentage of activity in the eluted fraction in comparison to the total starting activity. The fractions representing the ^47^Sc are black and the fractions representing the ^48^V are represented in solid gray.

**Figure 2:**
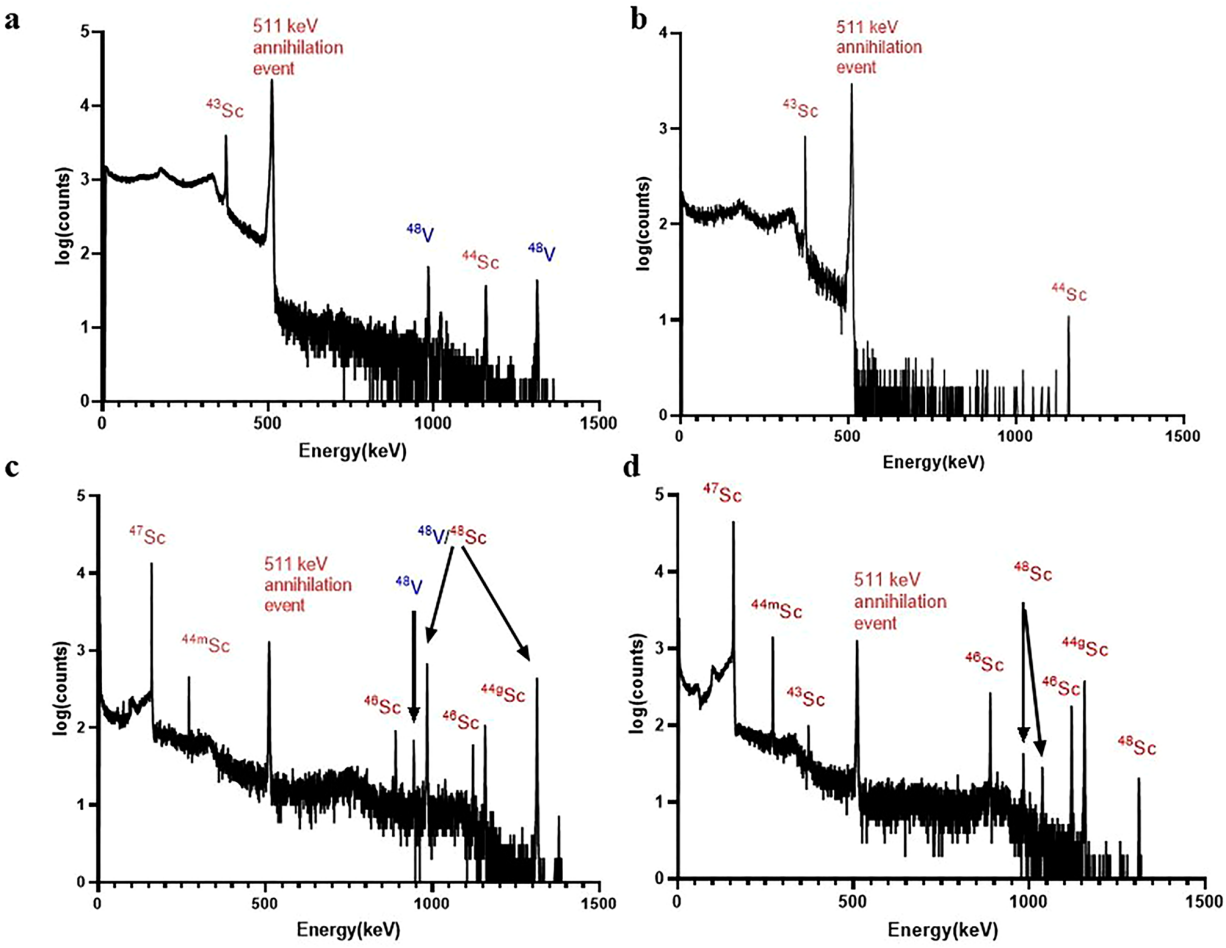
The gamma-ray spectra of the dissolved irradiated [^46^Ti]TiO_2_ target (**a**). The gamma-ray spectra of the purified ^43^Sc elution (**b**). The gamma-ray spectra of the dissolved irradiated [^50^Ti]TiO_2_ target (**c**). The gamma-ray spectra of the purified ^47^Sc elution (**d**). Each photopeak is labeled with the corresponding radionuclide.

Targets were readily dissolved in a pressurized PFA digestion vial before isolating the radioscandium nuclides from co-produced radiovanadium and the titanium starting material. The two-step dissolution process took a total of 3 h. A representative separation profile for ^47^Sc is depicted in Fig. 1 and supplemental information Table S7 show the results of the separation, where the total average ^43^Sc and ^47^Sc recovery yields were 91.7 ± 7.4% and 89.9 ± 3.9%, respectively, with the majority in the E4 fraction. The co-produced ^48^ V was removed before the elution of the ^43^Sc and ^47^Sc as shown in the gamma ray spectra in Fig. 2. The decay corrected activity at the end of bombardment for a 1.5 h run using ^46^Ti and a 4 h run using ^50^Ti was 510 MBq (13.8 mCi) for ^43^Sc and 52.17 MBq (1.42 mCi) for ^47^Sc (supplemental information Table S7).

The radionuclides were measured by gamma-ray spectroscopy using their characteristic γ-rays presented previously in Table S2. The identification of the radionuclides is shown in the HPGe spectra in Fig. 2. Each photopeak is labeled with their corresponding radionuclide. The spectra shown in panels a and c in Fig. 2 represent the dissolved target solution of either production whereas panels b and d are for the purified material.

**Figure 3:**
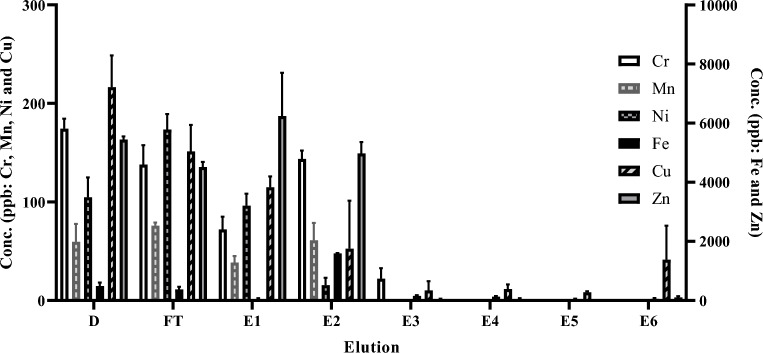
ICP-MS elemental analysis results for detectable trace metal contaminates found in the corresponding fractions (x-axis) during the purification of ^47^Sc. The element concentration range on the left y-axis is for Cr, Mn, Ni, and Cu. The element concentration range on the right y-axis is for Fe and Zn.

HPGe analysis was used to determine the final activity and the radionuclidic purity of ^43^Sc and ^47^Sc decay corrected to end of bombardment. Table 1 is the average activity produced of all radioscandium nuclides and the radionuclidic purity for [^46^Ti]TiO_2_ and [^50^Ti]TiO_2_ bombardments, decay corrected to end of bombardment. The quantification of the longer-lived ^46^Sc, ^47^Sc, ^48^Sc were measured at later time points (≥ 1 day for ^47^Sc and ^48^Sc and ≥ 4 weeks for ^46^Sc), after the shorter lived radioisotopes (^43^Sc and ^44^Sc) have decayed. The average radionuclidic purity for ^43^Sc from enriched targets was 98.8 ± 0.3%. The average radionuclidic purity for ^47^Sc from enriched targets was 91.5 ± 0.6%. ICP-MS was conducted to assess the elution and presence of trace metal contaminants including Cr, Mn, Ni, Fe, Cu, W, and Zn throughout the separation with results shown in Fig. 3. The following elements are shown to be removed before the elution of ^47^Sc: Cr, Mn, and Ni. Fe, Cu, W and Zn are shown to be significantly reduced before the product collection, < 15 ppb. All other elements measured were below the limit of detection (< 15 ppb for E3-6 and < 50 ppb for other eluted fraction). The elements monitored during analysis were based on the Certificate of Analysis in supplemental information Table S4.

**Table 1:** The calculated radionuclide purity of produced ^43^Sc and ^47^Sc at EOB.

Activity corrected to end of bombardment for the collected ^43^Sc: Fig. 2 (n = 3)							
Isotope	^43^Sc	^44g^Sc	^44m^Sc	^46^Sc	^47^Sc	^48^Sc	Total radioscandium
Activity MBq (mCi)	510 ± 22	5.19 ± 0.30	0.11 ± 0.01	0.02 ± 0.01	0.35 ± 0.04	< 0.01 ± 0.01	517.6 ± 25.5
	(13.7 ± 0.7)	(0.14 ± 0.01)	(< 0.01)	(< 0.01)	(0.01 ± 0.01)	(< 0.01)	(13.9 ± 0.7)
Percentage	98.8 ± 0.3	1.01 ± 0.1	0.02 ± < 0.01	< 0.01	0.07 ± < 0.01	< 0.01	100

Activity corrected to end of bombardment for the collected ^47^Sc: Fig. 2 (n = 3)							
Isotope	^43^Sc	^44g^Sc	^44m^Sc	^46^Sc	^47^Sc	^48^Sc	Total radioscandium
Activity MBq (mCi)	Decayed	1.63 ± 0.3 (0.04 ± 0.01)	1.63 ± 0.3	1.33 ± 0.05	52.2 ± 2.3	0.24 ± 0.08	57.4 ± 6.3
			(0.04 ± 0.01)	(0.04 ± 0.01)	(1.4 ± 0.17)	(< 0.01)	(1.5 ± 0.1)
Percentage	0	2.8 ± 0.6	2.8 ± 0.6	2.3 ± 0.4	91.5 ± 0.6	0.42 ± 0.6	100

Additionally, continued recycling of the target resulted in improved purity as both rinse fractions E2 and E3 were not included during the recycling process, therefore any impurities in those elutions were not reintroduced into the next cycle. This is shown in Table 2, where the trace metal analysis of purified ^47^Sc is compared to experiments where multiple-times recycled target material was used. All other elements monitored were below the limit of detection.

**Table 2:** Trace metal analysis of collected ^47^Sc from recycled ^50^Ti targets.

Element	Cycle 1 (ppb)	S.D	Cycle 4 (ppb)	S.D.	Cycle 6 (ppb)	S.D.
Cr	311	33.1	< 15^a^	–	< 15^a^	–
Mn	340	48.6	< 15^a^	–	< 15^a^	–
Fe	1433	130	194	25.4	< 15^a^	–
Ni	190	14.9	< 15^a^	–	< 15^a^	–
Cu	264	10.1	11.6	14.6	< 15^a^	–
Zn	431	25.1	47.6	28.6	27.8	12.5
W	10,171	37.9	53.1	44.5	31.6	1.81
Pb	2172	216	< 15^a^	–	< 15^a^	–

S.D., standard deviation.

^a^Below the limit of detection.

### Recycling yields

The average recovery from the recycling process was 96 ± 4% (n = 5), showing a high recovery of the target material. The recovery over several run cycles is presented in supplemental information Fig. S6 for [^46^Ti]Ti and [^50^Ti]Ti by ICP-MS analysis. Furthermore, Table 3 demonstrates the high target material recovery as the production yields from the same recycled targets, either [^46^Ti]TiO_2_ and [^50^Ti]TiO_2_, remain consistent.

**Table 3:** Production yields from single targets.

Production of ^43^Sc from a single enriched ^46^Ti target bombarded for 1.5 h			
Activity	Cycle 1	Cycle 2	Cycle 3
MBq	499	540	529
mCi	13.5	14.6	14.3

Production of ^47^Sc from a single enriched ^50^Ti target bombarded for 8–9 h			
Activity	Cycle 1^a^	Cycle 4	Cycle 8
MBq	110	85.1	84.7
mCi	2.97	2.3	2.29

^a^9 h bombardment.

### Apparent molar activity

The radioisotopes ^43^Sc or ^47^Sc were used to radiolabel DOTA at various concentrations with the % complex versus DOTA concentration represented in Table S6. An scan of an iTLC of a 100% radiochemical yield of [^47^Sc]Sc-DOTA and one scan with free [^47^Sc]Sc control is shown in Fig. S4. The apparent molar activity curve is shown in Fig. S7. The estimated apparent molar activity (AMA) was improved when the target material from later recycling cycles was used. The ^43^Sc AMA was 5.92 GBq/µmol (160.3 mCi/μmol) for the second target cycle and improved to 23.2 GBq/µmol (628 mCi/μmol) for the 6th cycle. The ^47^Sc AMA was 1.26 GBq/µmol (34.0 mCi/μmol) for 3rd cycle and was 3.39 GBq/µmol (91.7 mCi/μmol) for 6th cycle.

### Phantom imaging

**Figure 4:**
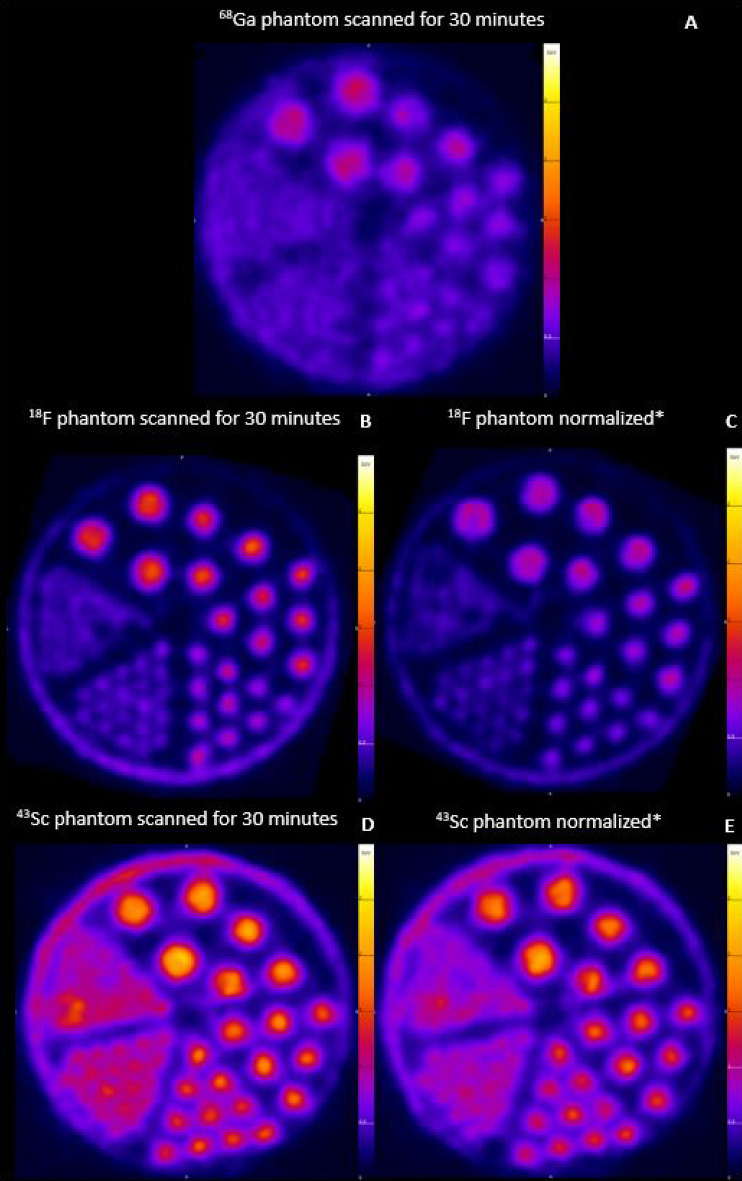
Phantom PET images of (**A**) ^68^Ga, (**B, C**) ^18^F, and (**D, E**) ^43^Sc. The ^68^Ga image contained 3.7 MBq (100 µCi), imaged for 30 min for 5.74E9 total decays. The ^18^F and ^43^Sc phantoms contained 3.7 ± 0.07 MBq (101 ± 2 µCi) and imaged for 30 min (**B** and **D**) or reconstructed to be normalized to ^68^Ga for same total decays (**C** and **E**). Images were further processed using Vivoquant (VivoQuant 4.0, Invicro Imaging Service and Software, Boston USA, vivoquant.com).

A phantom comparison was performed for two purposes: as a proof-of-concept comparison to reported literature and to assess the image quality of our produced ^43^Sc compared to the clinically used ^18^F and ^68^Ga and reported literature. All phantoms were prepared and imaged in the same manner, the ^18^F and ^43^Sc were reconstructed under two parameters: where the first reconstruction included the entire 30 min scan and the second reconstruction used shorter time frame (1693 s for ^18^F and 1614 s for ^43^Sc) for the same total decays of either isotope to match the total decays of the 30 min ^68^Ga scan. These images are shown in Fig. 4. A qualitative assessment of Fig. 4 suggests that ^43^Sc has a favorable imaging quality and slightly improved resolution over ^68^Ga for both reconstructions. Table 4 shows the numerical expression of the image difference using FWHM for all visibly distinguishable rods. The resulting FWHM corroborates the qualitative analysis of the resolution quality of these radionuclides, both for phantom scanned for the same time or reconstructed for the same total decays. The quantitative resolution results also illustrates that the order of resolution from highest to lowest is: ^18^F > ^43^Sc > ^68^Ga.

**Table 4:** The full-width half maximum for ^18^F, ^43^Sc and ^68^Ga^18^.

Radionuclide	Eβ^+^ average [keV]	Intensity	FWHM for 4.8 mm rod	FWHM for 4.0 mm	FWHM for 3.2 mm rod	FWHM for 2.4 mm rod	FWHM for 1.6 mm rod
^18^F (30 min scan)	250	96.7	4.55 ± 0.21	3.61 ± 0.18	2.61 ± 0.01	1.85 ± 0.01	1.43 ± 0.03
^18^F (Normalized to ^68^Ga)^a^			4.71 ± 0.05	3.68 ± 0.08	2.69 ± 0.07	2.00 ± 0.05	1.57 ± 0.04
^43^Sc (30 min scan)	476	88.1	4.40 ± 0.29	3.57 ± 0.08	2.76 ± 0.02	2.34 ± 0.11	1.53 ± 0.09
^43^Sc (Normalized to ^68^Ga)^a^			4.66 ± 0.03	3.59 ± 0.10	2.90 ± 0.07	2.44 ± 0.03	1.46 ± 0.04
^68^Ga (30 min scan)	830	88.9	4.84 ± 0.48	4.23 ± 0.10	3.84 ± 0.22	–	–

^a^Reconstructed to match the total decays of the ^68^Ga scan (5.74E9 total decays).

Both ^18^F and ^43^Sc transversal slice images were acquired from the same position. Both of the ^43^Sc scans were significantly different to the ^68^Ga scan at the 4.0 mm (p = 0.0004 for full 30 min scan and 0.003 for normalized) and 3.2 mm (p = 0.0009 for full 30 min scan and 0.001 for normalized). Both of the ^43^Sc scans were also significantly different from the ^18^F scan on the 2.4 mm rod (p = 0.034 for the 30-min scan and p =  < 0.0001 for the normalized). All other comparisons were not statistically significant.

## Discussion

The production of elementally matched theranostic pairs is highly sought after but commonly requires the use of expensive enriched material. Developing a target recycling method is often essential to offset the cost of enriched material. The work presented here demonstrates a reproducible purification and recycling route for ^43^Sc and ^47^Sc production starting from enriched ^46^Ti and ^50^Ti, respectively.

The radioactivity decay corrected to end of bombardment yields presented in Table S5 are in close agreement with the theoretical calculations and recent literature^17,26^. For ^43^Sc, 510.7 ± 21.8 MBq (13.8 ± 0.59 mCi) were produced, while the theoretical yield amount to 514.3 MBq (13.9 mCi) for a 1.5 h bombardment. In the case of ^47^Sc, 52.17 ± 2.3 MBq (1.3 ± 0.1 mCi) were produced versus 49.6 MBq (1.3 mCi) theoretical for a 4 h bombardment (n = 3 for each). The optimization of the separation proposed by Loveless et al. was achieved by adopting three improvements^17^. First, an additional wash of 3 mL of 1 M HNO_3_ was added, which improved the radioscandium purification by removing additional impurities (Cr, Fe, Cu and Zn are present in this elution as shown in Fig. 3) and reducing the final elution volume^17,26^. Next, the use of a SPE fritted tube instead of a BioRad column improved the time and reproducibility of the purification^17,26^. The SPE fritted tube columns were adapted for the use of a syringe pump that allowed for constant flow rates, which reduced flow rate variability. Lastly, the presence of HF in the dissolved target solution would decrease the radioscandium affinity to BDGA resin; thus, the TiO_2_ with NH_4_HF_2_ was heated for 2 h to ensure complete removal of the HF before addition of concentrated HCl^24,27^. These improvements allowed for shorter evaporation time as the collected volume was decreased from 10 to 3 mL for either the ^43^Sc or ^47^Sc while maintaining an effective separation of the radioscandium from titanium target material and ^48^V species^17^.

The irradiated targets for ^43^Sc were dissolved 30 min after end of bombardment to allow decay of the short-lived radio contaminates (such as ^13^N and ^15^O) to reduce the dose to personnel. The radiochemical purity of ^43^Sc was 98.8%, with ^44g^Sc, the other PET radionuclide, being the highest impurity at 1.01%. These results are in agreement with the reported radionuclidic purity from Domnanich et al.^26^.

The irradiated targets for ^47^Sc were dissolved the following day to allow for the decay of short-lived radiocontaminates and ^43^Sc and ^44g^Sc produced from other Ti isotopes present in the target material. The advantage of using enriched [^50^Ti]TiO_2_ can be observed in the HPGe analysis as the percent of ^47^Sc at end of bombardment is 91.5 ± 0.6% whereas reported radionuclidic ^47^Sc purity for ^nat^Ti targets, after which ^43^Sc and ^44g^Sc has substantially decayed, 28 h after end of bombardment, was 43%^17^.

The production of ^46^Sc, which is the radioscandium contaminate of concern as its half-life is 83 days and the longest lived radioscandium isotope, was determined to be < 2% of all total radioscandium for ^47^Sc production, an indication of the efficiency of using enriched target material. The production yields of ^46^Sc and other radioscandium isotopes could be reduced with the use of higher purity enriched [^50^Ti]TiO_2_. Ideally, the percentages of [^47^Ti]Ti and [^49^Ti]Ti should remain consistently low, as both titanium isotopes are routes for ^46^Sc production, as shown in supplemental information Fig. S2.

The ICP-MS results indicated the presence of trace metal contaminates within the target material, which is in alignment with the certificate of analysis shown in Table S4, with Cr, Mn at < 100 ppm and Fe, Cu, Zn, W, and Pb having some of the largest starting concentrations. The larger concentrations of Fe, Zn, and W are of concern as they will likely compete with radioscandium for complexation sites^28–30^. The separation is shown to be effective at removing these metals before eluting the desired radioscandium, as seen in Fig. 3. The majority of the contaminates were removed with the washes of 9 M HCl, 1 M and 7 M HNO_3_. The overall separation process indicates the removal of the trace metal contaminates and high recovery of Ti species in flow through and E1 via ICP-MS analysis.

The high target recovery yield of 96% provides a steady life cycle of the target material to help balance the cost of the enriched material. The percent recovery is also in accordance with the reported Domnanich et al.^26^ target recycling for ^nat^TiO_2_ (97.6%). Furthermore, the yields of activity produced in subsequent irradiations of the same target remained consistent, validating the constant quality of the target material up to eight target cycles The target recycling method here also resulted in a purification of the target material. As shown in the trace metal analysis of the purified ^47^Sc and the increasing AMA of both ^43^Sc and ^47^Sc, the target material was gradually purified during each cycle. This procedure shows high reproducibility of the target life cycle, from target collection to ^43^Sc and ^47^Sc purification and increased purity of the recycled target material after each use.

An additional characterization of the produced ^43^Sc and ^47^Sc is the measured AMA of the complex formation with DOTA. The separation and target purification from repeated recycles is shown to be effective for the removal of trace contaminants that may compete with ^43^Sc or ^47^Sc and is shown with the increased AMA after each target recycling^11^.

The PET images of the ^18^F, ^43^Sc and ^68^Ga were employed to validate ^43^Sc PET imaging resolution, considering two scenarios: 30 min static scan and scans reconstructed and normalized to the total decays of the ^68^Ga scan. The image resolution of ^43^Sc was quantified and compared to that of ^68^Ga, revealing a smaller FWHM at all visibly distinguishable rods. This suggests a higher resolution, particularly evident at the 3.2 mm rod diameter, which represents the smallest distinguishable rod on the ^68^Ga scan. Additionally, the resolution order remained consistent, with ^18^F having the highest resolution and the smallest FWHM value at each rod, while ^68^Ga exhibited the lowest resolution and largest FWHM values. The significant differences between the FWHM data confirms our hypothesis of decreasing resolution order of ^18^F > ^43^Sc > ^68^Ga. These results are in line with expectations, as higher positron energies result in a decreased resolution, which can already be observed by qualitative evaluation of the PET phantom images. The resulting 1.85 FWHM of ^18^F for the 2.4 mm rod is in agreement with literature values of 1.9 mm^31^. Comparing the FWHM of ^18^F and ^43^Sc on the next smaller rod size also corroborates that ^18^F has a higher resolution in comparison to ^43^Sc but as this resolution difference is demonstrated using a small animal PET scanner, it may have little impact in clinical practice using human scanners. These results indicate that ^43^Sc has favorable characteristics for PET imaging.

Future studies will continue the characterization of the proton induced nuclear reactions on enriched ^47^Ti for production of ^44^Sc and on enriched ^48^Ti for production of ^47^Sc. Although there are limited reports on the production of ^44g^Sc from titanium targets, the major challenge lies in the co-production of the metastable state ^44m^Sc (IT: t_1/2_: 58.6 h) during this production process^19^. However, there has been interest in utilizing ^44m^Sc as an in vivo generator for longer-lived targeting moieties like antibodies^32–34^.

Additionally, further analysis of the recycled TiO_2_ targets and proof-of-concept studies using ^43^Sc and ^47^Sc as a theranostic matched pair in targeted imaging and treatment will be explored. Further enhancement of the target design to increase production is desirable. The first improvement in the design revolves around augmenting the target thickness, thereby ensuring the capture of the entire excitation function under the energy of 24 MeV. The second design modification involves tailoring the target to accommodate higher energy cyclotrons (30 MeV). Theoretical predictions regarding ^47^Sc production, based on the 24 MeV design and the same enriched [^50^Ti]TiO_2_ and 4 h bombardment parameters in this work, would yield 5.8 mCi of ^47^Sc. The predicted radionuclidic purity for both ^46^Sc and ^47^Sc is 4.1% and 90.2%, respectively. The 30 MeV target design would increase the ^47^Sc yields to 11.6 mCi with a radionuclidic percentages of ^46^Sc and ^47^Sc at 6.2% and 90.1%, respectively. Although the percentage of ^46^Sc increases, it's noteworthy that the ^47^Sc yields nearly double while maintaining a comparable level of purity. Investigation of the cross sectional data for the ^50^Ti(p,p+α)^46^Sc at 30 MeV can help guide the ideal bombardment parameters, such as irradiating a 28 or 29 MeV, to decrease the ^46^Sc production. It’s important to acknowledge that the utilization of higher enrichment levels (> 83%) of [^50^Ti]TiO_2_ also holds potential. This is contingent upon the condition that the percentages of ^47^Ti and ^49^Ti remain equal or lower to reduce ^46^Sc and ^48^Sc when operating within energies of 30 MeV or below.

## Conclusions

This work demonstrates the feasibility of utilizing enriched [^46^Ti]TiO_2_ and [^50^Ti]TiO_2_ for the production of high purity ^43^Sc and ^47^Sc using 18 or 24 MeV protons. The enrichment of 83% [^50^Ti]TiO_2_ yielded 91% radionuclidic purity of ^47^Sc at the end of bombardment, after the decay of short lived ^43,44g^Sc while the 96% enriched [^46^Ti]TiO_2_ yielded 98% pure ^43^Sc at the end of bombardment. The reported separation technique removed radio and trace metal contaminates that would compete with complexation studies, results of which were verified by HPGe and ICP-MS analysis. The DOTA titration illustrates high molar activity with produced radioscandium that would allow the radiolabeling of other targeting compounds, encompassing a DOTA chelator, followed by subsequent in vivo and in vitro studies. The recycling method resulted in high recovery yields that would compensate for the high cost of target material and demonstrated purification of target material after each cycle.

### Supplementary Information

Supplementary Information.

## Data Availability

The datasets generated during and/or analyzed during the current study are available from the corresponding author on reasonable request.
