# Supplementary material for: Production and purification of 43Sc and 47Sc from enriched [46Ti]TiO2 and [50Ti]TiO2 targets
Source: Sci Rep. 2023 Dec 19;13:22683. doi: 10.1038/s41598-023-49377-7 (PMC10730517; doi:10.1038/s41598-023-49377-7)
Supplement: Supplementary file 1 — Supplementary Information. [file 41598_2023_49377_MOESM1_ESM.docx]

Supplemental Information for: Production and purification of ^43^Sc and ^47^Sc from enriched [^46^Ti]TiO_2_ and [^50^Ti]TiO_2_ targets

Shelbie J. Cingoranelli^a,b^, Jennifer L. Bartels^b^, Pavithra H. A. Kankanamalage^c^, C. Shaun Loveless^b^, David A. Rotsch^c,d^ Suzanne E. Lapi^a,b*^

^a^Department of Chemistry, University of Alabama at Birmingham

^b^Department of Radiology, University of Alabama at Birmingham

^c^Physics Division, Argonne National Laboratory

^d^Radioisotope Science and Technology Division, Oak Ridge National Laboratory

* Corresponding author; E-mail: lapi@uab.edu. Telephone (205) 975-8689. Address 1924 6th Ave. S., WTI 310F, Birmingham, AL 35244.

Table of Contents

- 1. Relevant characteristics of ^68^Ga, ^177^Lu and ^43,44g,47^Sc
  2. Radionuclides produced from proton bombardment of ^nat^Ti targets
  3. Isotopic purity of enriched materials
  4. Certificate of Analysis of enriched materials
  5. Cross section measurements of ^46^Ti(p,α)^43^Sc and ^50^Ti(p,α)^47^Sc
  6. Cross sections of proton induced reactions on ^nat^Ti
  7. Schematic of the radioscandium purification method
  8. Inductively Coupled Plasma-Mass Spectrometry
  9. Radiolabeling conditions and iTLC
  10. Phantom dimension
  11. Average radioactivity per elution of purification
  12. Enriched ^46^Ti and ^50^Ti recovery
  13. The apparent molar activity curves for [^43^Sc]Sc-DOTA and [^47^Sc]Sc-DOTA
  14. Relevant characteristics of ^68^Ga, ^177^Lu and ^43,44g,47^Sc

**Table S1:** Characteristics of the radioisotopes of Ga, Lu and Sc.^1,2^

| Radioisotope | Half-life | [β+/-] mean energy (keV) | Additional γ (branching ratio %) |
| --- | --- | --- | --- |
| ^68^Ga | 68 min | 829.5 (88.9%): [β ^+^] | 1077.3 (3.22) |
| ^43^Sc | 3.9 h | 476.0 (88.1%): [β ^+^] | 372.9 (22.5) |
| ^44g^Sc | 3.9 h | 632.0 (94.3%): [β ^+^] | 1157.0 (99.9) |
| ^177^Lu | 6.6 d | 133.6 (100.0%): [β ^-^] | 112.9 (6.23) & 208.4 (10.4) |
| ^47^Sc | 3.3 d | 162.0 (100.0%): [β ^-^] | 159.3 (68.3) |

- 1. Radionuclides produced from proton bombardment of ^nat^Ti targets

**Table S2:** Products from proton irradiation of ^nat^Ti.^1-3^

| **Isotope** | **Nuclear reaction** | **Half-life** | **Decay mode** | **Key gamma rays** |
| --- | --- | --- | --- | --- |
| ^43^Sc | ^46^Ti(p,α)^43^Sc | 3.9 h | β^+^ | 372 (22.5%) |
| ^44g^Sc | ^47^Ti(p,α)^44g^Sc | 3.9 h | β^+^ | 1157 (99.9%) |
| ^44m^Sc | ^47^Ti(p,α)^44m^Sc | 58.6 h | IT : 98.8%  ε: 1.2% | 271 (86.74%) |
| ^46^Sc | ^47^Ti(p,2p)^46^Sc  ^49^Ti(p,α)^46^Sc  ^50^Ti(p,n+α)^46^Sc | 83.7 d | β^-^ | 889 (99.98%)  1120(99.98%) |
| ^47^Sc | ^48^Ti(p,2p)^47^Sc  ^50^Ti(p,α)^47^Sc | 3.9 d | β^-^ | 159 (68.3%) |
| ^48^Sc | ^49^Ti(p,2p)^48^Sc | 48.6 h | β^+^ | 175 (7.48%) |
|  |  |  |  | 983 (100%) |
|  |  |  |  | 1037 (97.6%) |
|  |  |  |  | 1212 (2.38%) |
|  |  |  |  | 1312 (100%) |
| ^48^V | ^48^Ti(p,n)^48^V  ^49^Ti(p,2n)^48^V | 15.9 d | β^+^ | 944 (7.87%)  983 (98.98%) |
|  |  |  |  | 1. (98.2%) |

c. Isotopic purity of enriched materials

| **Table S3:** The isotopic enrichment of [^46^Ti]TiO_2_ and [^50^Ti]TiO_2_. All materials were provided by the National Isotope Development Center, USA. | | |
| --- | --- | --- |
| **Enrichment of ^x^TiO_2_** | | |
| ^X^Ti Isotope | Isotopic Abundance in [^46^Ti]TiO_2_ | Isotopic Abundance in [^50^Ti]TiO_2_ |
| 46 | ***96.84*** | 1.69 |
| 47 | 0.5 | 1.29 |
| 48 | 2.33 | 12.51 |
| 49 | 0.16 | 1.41 |
| 50 | 0.16 | ***83.1*** |

1. Isotopic purity of enriched materials

**Table S4:** The Certificates of Analysis (CoA) of [^46^Ti]TiO_2_ and [^50^Ti]TiO_2_, measured in ppm. Both the materials and the CoA were provided by the National Isotope Development Center, USA.

| Element: ppm | ^46^Ti | ^50^Ti | Element: ppm | ^46^Ti | ^50^Ti | Element: ppm | ^46^Ti | ^50^Ti |
| --- | --- | --- | --- | --- | --- | --- | --- | --- |
| Ag | <50 | <50 | Mg | <100T | <100T | Lanthanides and Actinides | | |
| Al | <100T | <200T | Mn | <100 | <100 | Ce | <1000 | <1000 |
| Au | <500 | <500 | Mo | <100 | <100 | Dy | <1000 | <1000 |
| B | <100T | <100T | Na | <=100 | 1000 | Er | <50 | <50 |
| Ba | <100 | <100 | Nb | <500 | <500 | Eu | <50 | <50 |
| Be | <10 | <10 | Ni | <100 | <200 | Gd | <200 | <200 |
| Bi | <200 | <200 | Pb | <200 | <200 | Ho | <500 | <500 |
| Ca | <100 | 100 | Pt | <500 | <500 | La | <200 | <200 |
| Cd | <500 | <500 | Rb | <200 | <20 | Lu | <50 | <50 |
| Co | <100 | <200 | Sb | <500 | <500 | Nd | <500 | <500 |
| Cr | <100 | <200 | Si | 300 | <=100 | Pr | <500 | <500 |
| Cs | <500 | <500 | Sn | <100 | <200 | Sm | <500 | <500 |
| Cu | 200 | 200 | Sr | <100 | <100 | Tb | <500 | <500 |
| Fe | <100 | 500 | Ta | <500 | <500 | Y | <50 | <50 |
| Ga | <200 | <200 | Te |  | <500 | Yb | <20 | <20 |
| Ge | <200 | <200 | Ti | M | M | Tm | <500 | <500 |
| Hf | <500 |  | V | <100 | <200 |  |  |  |
| Hg | <500 | <500 | W | <500 | 1000 |  |  |  |
| In | <500 |  | Zn | <2000 | <500 |  |  |  |
| K | <100 | <100 | Zr | <200T | <200 |  |  |  |
| Li | BUFFER* | <50 |  |  |  |  |  |  |

*Was added during analysis. No values were obtained.

Where: M is major; T is trace; <= less than/equal; < is less than, nd is not detected; and elements listed without values were not detected or less than 10 ppm.

**
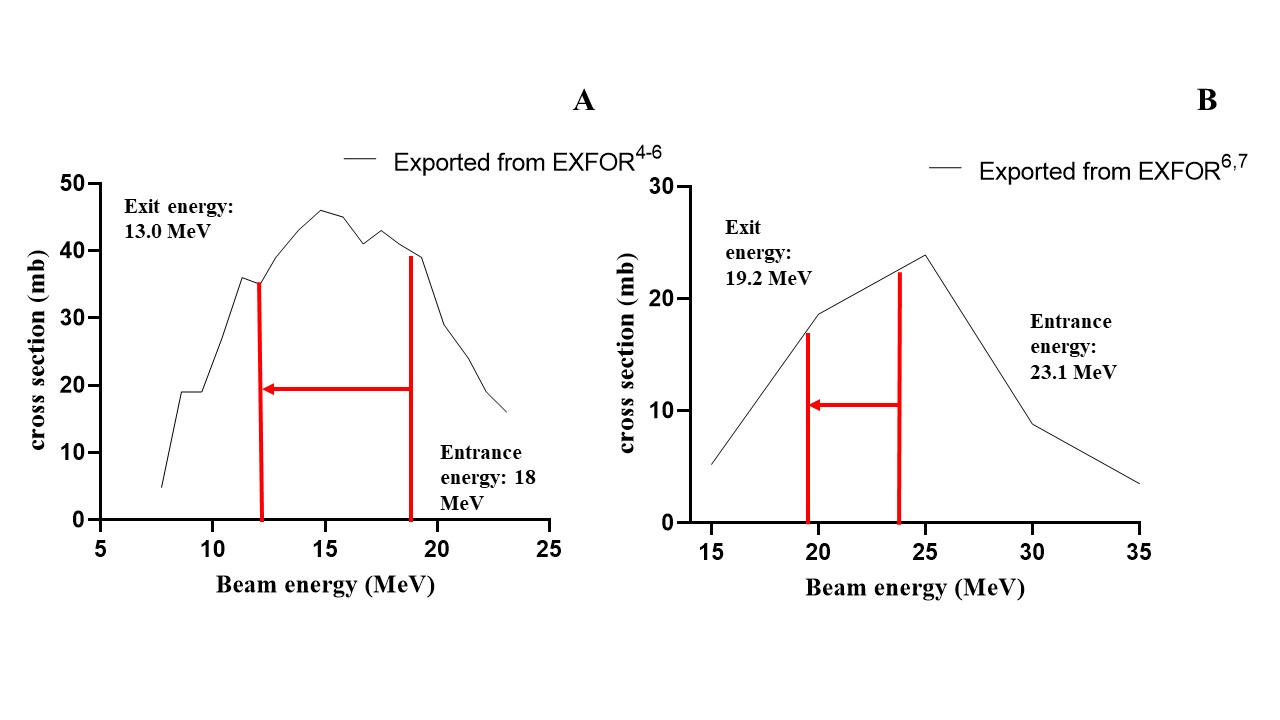
**e. Cross section measurements of ^46^Ti(p,α)^43^Sc and ^50^Ti(p,α)^47^Sc

**Figure S1:** Excitation function for ^46^Ti(p,α)^43^Sc with the red lines indicating the relevant energy range during bombardment at 18 MeV (A).^4-6^. Excitation function for ^50^Ti(p,α)^47^Sc with the red lines indicating the relevant energy range during bombardment at 24 MeV(B).^6,7^

f. Cross sections of nuclear reactions from proton irradiation on ^nat^Ti.


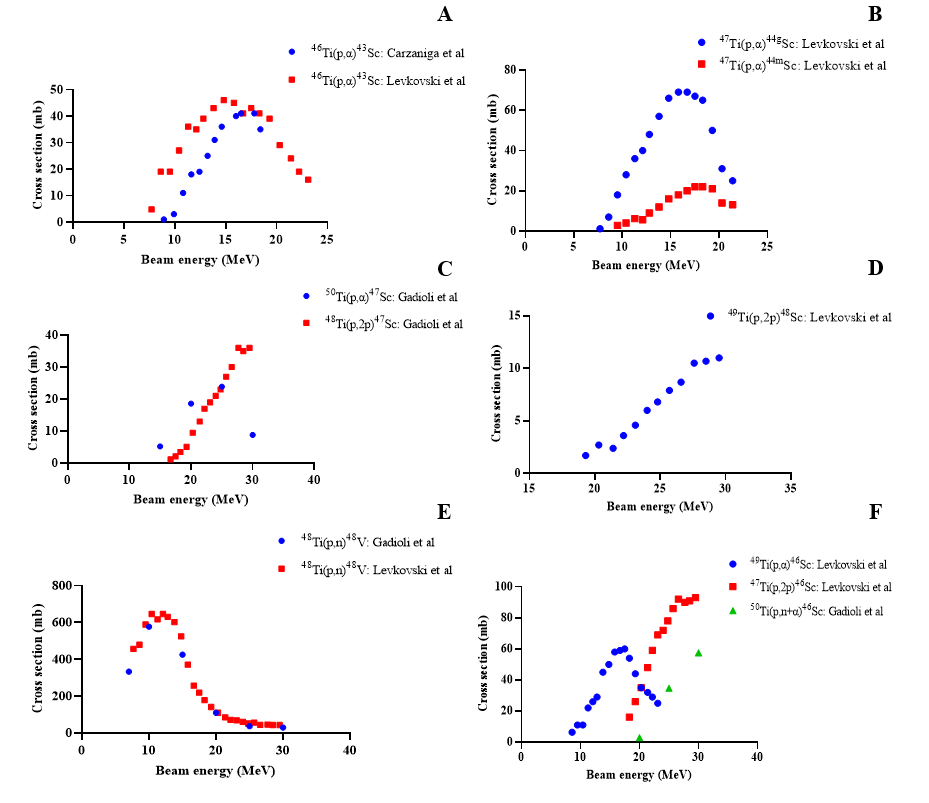


**Figure S2**: Representative cross sections of nuclear reactions occurring during 0-30 MeV proton irradiations on ^nat^Ti targetes.^4,5,7^ Cross sections were imported from EXFOR and used to calculate the theoretical yields during irradiations as well as determine the optimal bombardment parameters. (A) ^43^Sc production via ^46^Ti(p,α) ^43^Sc. (B) ^44g/m^Sc production via ^47^Ti(p,α)^44g/m^Sc. (C) The production of ^47^Sc via ^48^Ti(p,2p)^47^Sc and ^50^Ti(p,α)^47^Sc. (D) ^48^Sc production via ^49^Ti(p,2p)^48^Sc. (E) ^48^V production via ^48^Ti(p,n)^48^V. (F) Production of ^46^Sc from ^49^Ti(p,α)^46^Sc, ^47^Ti(p,2p)^46^Sc and ^50^Ti(p,n+α)^46^Sc.

g. Schematic of the radioscandium purification method


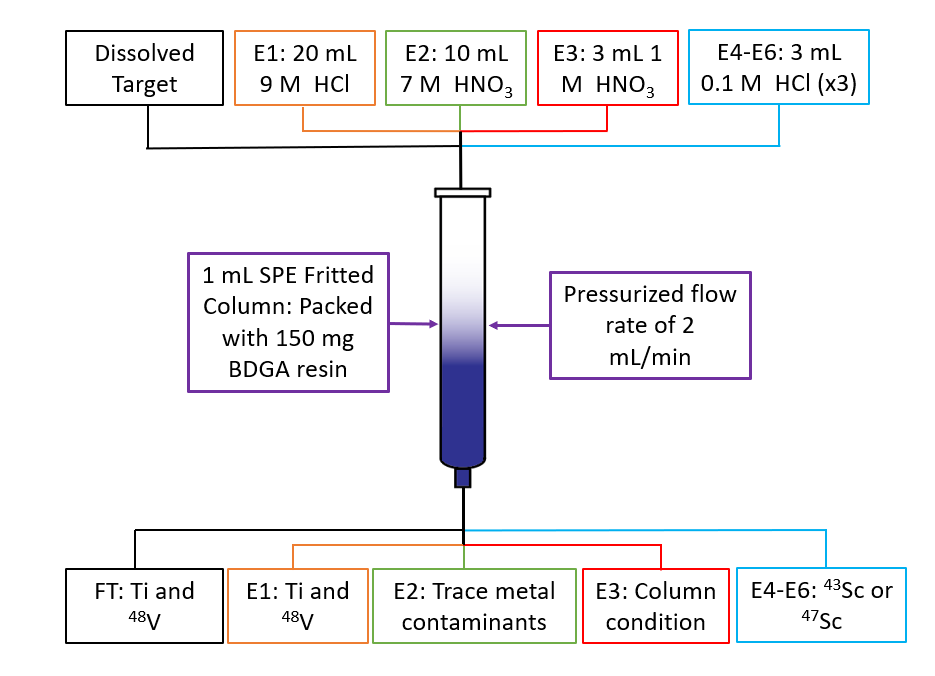


**Figure S3:** Optimized radioscandium purification method. Flow Through (FT) is the dissolved target solution. E1-E3 are three different washes for the removal of impurities as well as column conditioning. E4-E6 are the radioscandium elution fractions.

1. Inductively Coupled Plasma – Mass Spectrometry (ICP-MS)

**Table S5**: The list of monitored elements for each target material used and separation performed.

|  | | | | |
| --- | --- | --- | --- | --- |
| Transition metals | | Lanthanides and Actinides | | Other |
| Sc | Fe | Ce | Dy | Al |
| Ti | Co | Pr | Ho | Ge |
| V | Ni | Nd | Er | Ga |
| Cr | Cu | Sm | Tm | Pb |
| Mn | Zn | Eu | Yb | Ca |
| Zr | W | Gd | Lu |  |
| Nb | Ta | Tb | U |  |

1. Radiolabeling conditions and iTLC

1,4,7,10-tetraazacyclododecana-1,4,7,10-tetraacetic acid (DOTA) was dissolved in 0.25 M ammonium acetate buffer, pH 4.5, to produce a 10 mg/mL concentration stock. This was further diluted in the same buffer to final volumes of 100 µL with DOTA concentrations provided in Table S6. Subsequently, either ^43^Sc, 3.7 MBq (100 µCi) or ^47^Sc, 2.3 MBq (62.5 µCi) was added to the vials, which were incubated at 95 ^o^C for 30 min. The resulting samples were spotted onto iTLC-SG paper (Agilent) and the paper was developed in 1 M citric acid. The iTLC strips were analyzed on an AR-2000 instrument using Winscan software (Eckert and Ziegler). In the analysis, the DOTA complex exhibited an RF of 0.65, while free ^4x^Sc migrated with the solvent front, as illustrated in **Figure S4**. To determine the apparent molar activity, the EC50 value of the DOTA titration was used to calculate the µmol for total binding. The average activity used for each vial, adjusted for decay, was then divided by this value.

| **Table S6**: The serial dilution of DOTA. | | | |
| --- | --- | --- | --- |
| Vial | Volume buffer | Serial dilution volume | Final concentration of DOTA(nmol) |
| 1 | 135 | 15 µL from 10 mg/mL stock | 247 |
| 2 | 100 | 50 µL from 1 | 82.4 |
| 3 | 100 | 50 µL from 2 | 27.5 |
| 4 | 100 | 50 µL from 2 | 9.16 |
| 5 | 100 | 50 µL from 4 | 3.05 |
| 6 | 100 | 50 µL from 5 | 1.02 |
| 7 | 100 | 50 µL from 6 | 0.34 |
| 8 | 100 | 50 µL from 7 | 0.11 |
| 9 | 100 | 50 µL from 8 | 0.04 |
| 10 | 100 | 50 µL from 9 | 0.01 |
| Discard | 0 | 50 µL from 10 | 0.00 |


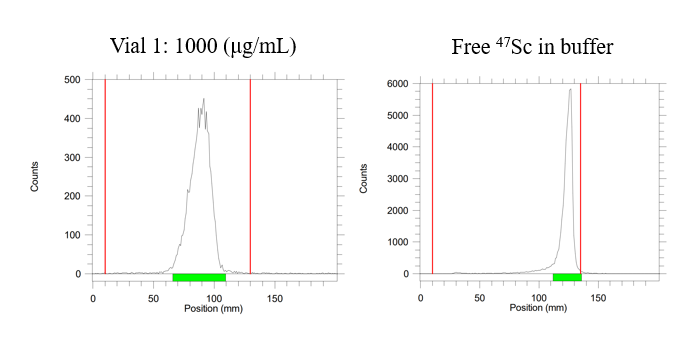


**Figure S4**: The radio-iTLC of [^47^Sc]Sc-DOTA at 0.24 μmol DOTA concentration, R_F_ = 0.65 (A). The radio-iTLC of free [^47^Sc]ScCl_3_ in 0.1 M HCl, R_F_ = 1. (B) The red lines mark the origin at 10 mm and solvent front at 140 mm.

1. PET phantom dimensions


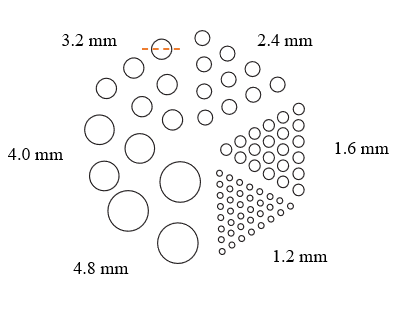


**Figure S5**: Schematic of the rod alignment and rod diameter of the phantom used for PET resolution comparison of three radionuclides: ^18^F, ^43^Sc and ^68^Ga. The dashed line represents intensity plot line that was drawn to gather the Full-width at half-maximum data.

A phantom composed of PMMA with rod diameters of 1.2, 1.6, 2.4, 3.2, 4.0 and 4.8 mm with a height of 3.4 cm, with the alignment represented in figure S5. The insert diameter was 4.4 cm, the cylinder outside diameter was 5 cm, the cylinder inside diameter was 4.5 cm, and the cylinder inside height was 6.3 cm.

1. Average activity per elution of purification

**Table S7:** Average activity per elution for a 1.5 hour bombardment (n=3) on [^46^Ti] and 4 hour bombardment (n=3) on [^50^Ti], where DT stands for Dissolved Target and FT stands for Flow-through

| **Target** | [^46^Ti]TiO_2_ | | | | [^50^Ti]TiO_2_ | | | |
| --- | --- | --- | --- | --- | --- | --- | --- | --- |
| **Elutions** | ^43^Sc MBq (mCi) | SD | ^48^V in MBq (μCi) | SD | ^47^Sc MBq (mCi) | SD | ^48^V in MBq (μCi) | SD |
| **DT** | 510 | 21.8  (0.59) | 2.51 | 0.29  (7.7) | 52.17 | 2.35  (0.06) | 8.29 | 4.29  (116) |
|  | (13.8) |  | (67.6) |  | (1.42) |  | (224) |  |
| **FT** | 27.8 | 6.29  (0.17) | 0.93 | 0.09  (2.3) | 1.95 | 0.72  (0.02) | 5.76 | 3.48  (94.1) |
|  | (0.75) |  | (25) |  | (0.05) |  | (156) |  |
| **E1** | 4.07 | 2.59  (0.07) | 1.55 | 0.58  (15.7) | 0.18 | 0.14  (<0.01) | 1.69 | 0.132  (8.85) |
|  | (0.11) |  | (41.9) |  | (<0.01) |  | (45.5) |  |
| **E2** | 15.17 | 1.48  (0.4) | 1.18 | 0.22  (5.8) | 0.26 | 0.34  (0.01) | 2.04 | 0.24  (6.62) |
|  | (0.41) |  | (32) |  | (0.01) |  | (55.1) |  |
| **E3** | 1.85 | 0.74  (0.02) | 0 | 0  0 | 0.08 | 0.02  (<0.01) | 0.04 | 0.34  (0.94) |
|  | (0.05) |  | 0 |  | (<0.01) |  | (0.09) |  |
| **E4** | 473 | 4.44  (0.12) | 0 | 0  0 | 47.8 | 6.3  (0.11) | 0 | 0  0 |
|  | (12.8) |  | 0 |  | (1.27) |  | 0 |  |
| **E5** | 3.7 | 2.96  (0.08) | 0 | 0  0 | 1.32 | 0.54  (0.01) | 0 | 0  0 |
|  | (0.1) |  | 0 |  | (0.04) |  | 0 |  |
| **E6** | 4.81 | 0  0 | 0 | 0  0 | 0.19 | 0.08  (<0.01) | 0 | 0  0 |
|  | (0.13) |  | 0 |  | (0.01) |  | 0 |  |

1. Enriched ^46^Ti and ^50^Ti recovery


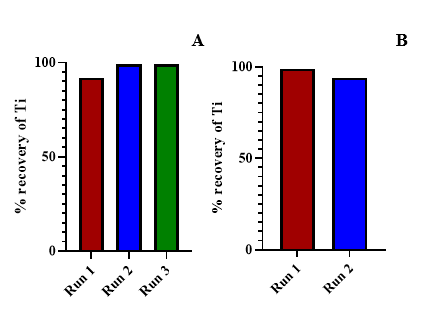


**Figure S6:** The percent recovery of the enriched [^50^Ti]TiO_2_ target for each run cycle, consisting of bombardment, processing, and recycling (A). The percent recovery for enriched [^46^Ti]TiO_2_ target for each run cycle, consisting of bombardment, processing, and recycling (B).

1. The apparent molar activity curves for [^43^Sc]Sc-DOTA and [^47^Sc]Sc-DOTA

**
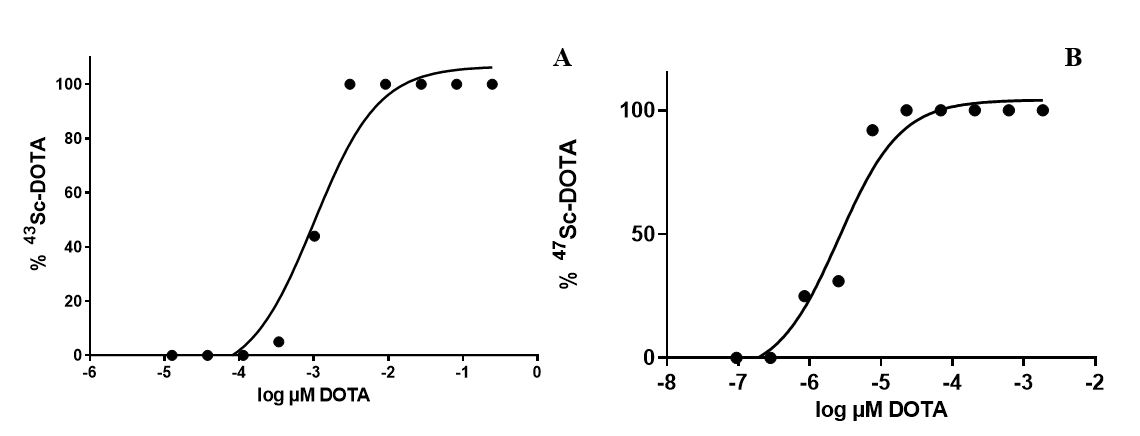
**

**Figure S7:** The apparent molar activity curve for the complexation of [^43^Sc]Sc-DOTA, where the complexation is represented by % complex as a function of the concentration of DOTA (A). The apparent molar activity curve for the complexation of [^47^Sc]Sc-DOTA, where the complexation is represented by % complex as a function of the concentration of DOTA (B).

Reference

1 NuDat 2.8. Brookhaven National Laboratory website. https://nndc.bnl.gov/nudat2/. Data extracted June 2, 2021. Accessed January 1, 2022

2 Chernysheva, M. *et al.* Accelerator Production of Scandium Radioisotopes: Sc-43, Sc-44, and Sc-47. *Curr Radiopharm* **14**, 359-373, doi:10.2174/1874471014999210112205535 (2021).

3 Loveless, C. S. *et al.* Cyclotron Production and Separation of Scandium Radionuclides from Natural Titanium Metal and Titanium Dioxide Targets. *Journal of Nuclear Medicine* **62**, 131-136, doi:10.2967/jnumed.120.242941 (2021).

4 Carzaniga, T. S. *et al.* Measurement of (43)Sc and (44)Sc production cross-section with an 18MeV medical PET cyclotron. *Appl Radiat Isot* **129**, 96-102, doi:10.1016/j.apradiso.2017.08.013 (2017).

5 Levkovski, V. N. Cross sections of medium mass nuclide activation (A=40-100) by medium energy protons and alpha-particles (E=10-50 MeV). *Atomnaya Energiya* **69**, 180 (1991).

6 Otuka, N. *et al.* Towards a More Complete and Accurate Experimental Nuclear Reaction Data Library (EXFOR): International Collaboration Between Nuclear Reaction Data Centres (NRDC). *Nuclear Data Sheets* **120**, 272-276, doi:<https://doi.org/10.1016/j.nds.2014.07.065> (2014).

7 Gadioli, E., Gadioli Erba, E., Hogan, J. J. & Burns, K. I. Emission of alpha particles in the interaction of 10–85 MeV protons with48,50Ti. *Zeitschrift für Physik A Atoms and Nuclei* **301**, 289-300, doi:10.1007/BF01421692 (1981).
